# The Sodium Sialic Acid Symporter From *Staphylococcus aureus* Has Altered Substrate Specificity

**DOI:** 10.3389/fchem.2018.00233

**Published:** 2018-07-04

**Authors:** Rachel A. North, Weixiao Y. Wahlgren, Daniela M. Remus, Mariafrancesca Scalise, Sarah A. Kessans, Elin Dunevall, Elin Claesson, Tatiana P. Soares da Costa, Matthew A. Perugini, S. Ramaswamy, Jane R. Allison, Cesare Indiveri, Rosmarie Friemann, Renwick C. J. Dobson

**Affiliations:** ^1^School of Biological Sciences, University of Canterbury, Christchurch, New Zealand; ^2^Biomolecular Interaction Centre, University of Canterbury, Christchurch, New Zealand; ^3^Department of Chemistry and Molecular Biology, University of Gothenburg, Gothenburg, Sweden; ^4^Centre for Antibiotic Resistance Research, University of Gothenburg, Gothenburg, Sweden; ^5^Unit of Biochemistry and Molecular Biotechnology, Department DiBEST (Biologia, Ecologia, Scienze della Terra), University of Calabria, Arcavacata di Rende, Italy; ^6^Department of Biochemistry and Genetics, La Trobe Institute for Molecular Science, La Trobe University, Melbourne, VIC, Australia; ^7^The Institute for Stem Cell Biology and Regenerative Medicine, Bangalore, India; ^8^Centre for Theoretical Chemistry and Physics, Institute of Natural and Mathematical Sciences, Massey University, Auckland, New Zealand; ^9^Maurice Wilkins Centre for Molecular Biodiscovery, University of Auckland, Auckland, New Zealand; ^10^Department of Biochemistry and Molecular Biology, Bio21 Molecular Science and Biotechnology Institute, University of Melbourne, Parkville, VIC, Australia

**Keywords:** antibiotic resistance, sialic acids, SiaT, sodium solute symporter, *Staphylococcus aureus*

## Abstract

Mammalian cell surfaces are decorated with complex glycoconjugates that terminate with negatively charged sialic acids. Commensal and pathogenic bacteria can use host-derived sialic acids for a competitive advantage, but require a functional sialic acid transporter to import the sugar into the cell. This work investigates the sodium sialic acid symporter (SiaT) from *Staphylococcus aureus* (*Sa*SiaT). We demonstrate that *Sa*SiaT rescues an *Escherichia coli* strain lacking its endogenous sialic acid transporter when grown on the sialic acids *N*-acetylneuraminic acid (Neu5Ac) or *N*-glycolylneuraminic acid (Neu5Gc). We then develop an expression, purification and detergent solubilization system for *Sa*SiaT and demonstrate that the protein is largely monodisperse in solution with a stable monomeric oligomeric state. Binding studies reveal that *Sa*SiaT has a higher affinity for Neu5Gc over Neu5Ac, which was unexpected and is not seen in another SiaT homolog. We develop a homology model and use comparative sequence analyses to identify substitutions in the substrate-binding site of *Sa*SiaT that may explain the altered specificity. *Sa*SiaT is shown to be electrogenic, and transport is dependent upon more than one Na^+^ ion for every sialic acid molecule. A functional sialic acid transporter is essential for the uptake and utilization of sialic acid in a range of pathogenic bacteria, and developing new inhibitors that target these transporters is a valid mechanism for inhibiting bacterial growth. By demonstrating a route to functional recombinant *Sa*SiaT, and developing the *in vivo* and *in vitro* assay systems, our work underpins the design of inhibitors to this transporter.

## Introduction

Mammalian cell surfaces are decorated with complex glycoconjugates, such as glycoproteins and glycolipids. Found at the terminal non-reducing positions of these cell-surface glycoconjugates are negatively charged sialic acids, which mediate a diverse array of cellular interactions, recognition and adhesion.

Sialic acids comprise a large family of nine-carbon acidic monosaccharides, the most common of which is *N*-acetylneuraminic acid (Neu5Ac). While Neu5Ac is ubiquitously synthesized, the closely related sialic acid, *N*-glycolylneuraminic acid (Neu5Gc) is not. Although Neu5Ac and Neu5Gc sialic acids are widely expressed on mammalian tissues, human cells do not synthesize Neu5Gc. This is because humans have an inactivating mutation in the gene encoding CMP-*N*-acetylneuraminic acid hydroxylase, the rate-limiting enzyme for the generation of Neu5Gc in the cells of other mammals (Varki, 2001).

Mammalian commensal and pathogenic bacteria that colonize sialic acid rich tissues, such as the respiratory or gastrointestinal tract, have evolved mechanisms to use host-derived sialic acids for a competitive advantage; this suggests a link between sialic acid uptake/utilization and survival *in vivo* (Almagro-Moreno and Boyd, 2009). Some bacteria, such as *Haemophilus influenzae* (Vimr et al., 2000; Bouchet et al., 2003), and *Neisseria meningitides* (Vimr et al., 2004) incorporate sialic acid into their cell surface macromolecules to trick the host's innate immune response. Others, such as *Escherichia coli* (Vimr and Troy, 1985; Chang et al., 2004), *Staphylococcus aureus* (Olson et al., 2013), and *Vibrio vulnificus* (Jeong et al., 2009) use a suite of enzymes (North et al., 2013, 2014a,b, 2016) to degrade sialic acids as a source of carbon, nitrogen and energy. Notably, *H. influenzae* also metabolizes sialic acids in this way, and must make a metabolic decision between cell surface sialylation and sialic acid degradation (Vimr et al., 2000).

Bacteria that import sialic acids have evolved multiple mechanisms of transport across the cytoplasmic membrane. To date, four unique transporter families have been recognized, including those from the ATP binding cassette (ABC) (Post et al., 2005), tripartite ATP-independent periplasmic (TRAP) (Allen et al., 2005), major facilitator superfamily (MFS) (Vimr and Troy, 1985), and sodium solute symporter (SSS) (Severi et al., 2010; Wahlgren et al., 2018) transporter families (North et al., 2017). Whilst most bacteria possess only one type of sialic acid transporter, there are a few exceptions that are predicted to express two family types (Severi et al., 2010). It is not understood why these organisms produce more than one type of sialic acid transporter, but it is possible that they import sialic acid derivatives that are known in biological contexts.

Developing novel inhibitors that target bacterial sialic acid transporters may be a valid mechanism for inhibiting bacterial growth—several lines of evidence support this. It has been shown that a dedicated and functional sialic acid membrane transporter is required for the uptake of sialic acids (Vimr and Troy, 1985; Severi et al., 2005, 2010). Moreover, *in vivo* mouse studies demonstrate that sialic acid uptake and utilization is essential for colonization and persistence in a range of pathogenic bacteria (Chang et al., 2004; Almagro-Moreno and Boyd, 2009; Jeong et al., 2009; Pezzicoli et al., 2012). Knocking out the respective sialic acid transporter genes in *Salmonella enterica serovar* Typhirium and *Clostridium difficile* impairs outgrowth during post-antibiotic expansion (Ng et al., 2013), and *E. coli* during intestinal inflammation (Huang et al., 2015). Humans readily synthesize the Neu5Ac type of sialic acid and have dedicated membrane transporters to deploy it onto their surface. These share little homology to the bacterial transporters (North et al., 2017) so inhibitors to the bacterial transporters may not be toxic.

Recently, we determined the high-resolution outward-facing, and open, substrate-bound structure of the SiaT sialic acid transporter from *Proteus mirabilis* (*Pm*SiaT) (Wahlgren et al., 2018). SiaT transporters belong to the SSS family. *Pm*SiaT adopts the LeuT-fold with Neu5Ac bound near the center of the protein, and two Na^+^ ions for transport.

This work investigates the SiaT sodium sialic acid symporter from *S. aureus* (*Sa*SiaT). We demonstrate that *Sa*SiaT can be purified and stably occupies a monomeric oligomeric state. We characterize the functionality of *Sa*SiaT with two different sialic acids, and the kinetics of sialic acid membrane transport.

## Materials and methods

### Molecular biology techniques

The *S. aureus* (strain RF122*) siaT* (Accession AJ938182.1) gene was codon optimized for *E. coli* (GeneArt, ThermoFischer Scientific; Supplementary Figure 1). For purification of recombinant protein and functional studies, *siaT* was amplified by PCR using *Sa_siaT*-F1 and *Sa_siaT*-R1 oligonucleotides (Supplementary Table 1) and cloned into the pWarf(-) (Hsieh et al., 2010) vector using the In-Fusion HD Cloning Kit (Clontech). The pWarf(-) vector carries a C-terminal human rhinovirus 3C protease (HRV3C) cleavage site followed by a green fluorescence protein (GFP)-tag and an 8 × histidine (His)-tag. The amplified fragment was cloned into pWarf(-) digested with the BamHI (3′) and XhoI (5′) restriction enzymes to generate pWarf(-)*Sa_siaT* with kanamycin resistance. This was transformed into Stellar™ Competent Cells (Clontech), purified using the DNA-Spin™ Plasmid DNA Purification Kit (iNtRon Biotechnology), and verified by DNA sequencing (Eurofins).

For bacterial growth experiments, *siaT* was amplified by PCR using *Sa_siaT*-F2 and *Sa_siaT*-R2 oligonucleotides (Supplementary Table 1) and cloned into the low-copy vector pJ422-01 also using the In-Fusion HD Cloning Kit. The amplified fragment was cloned into pJ422-01 digested with the EcoR1 (3′) and Nde1 (5′) restriction enzymes to generate pJ422-01*Sa_siaT* with Zeocin™ resistance. This was transformed into Stellar™ Competent Cells (Clontech), purified using the DNA-Spin™ Plasmid DNA Purification Kit (iNtRon Biotechnology), and verified by DNA sequencing (Genetic Analysis Service, University of Otago). The pJ422-01*Sa_siaT* plasmid was subsequently transformed into the *E. coli* JW3193 Δ*nanT* strain [NBRP (NIG, Japan): *E. coli*] (Baba et al., 2006) generating the complementation strain *E. coli* JW3193 Δ*nanT-siaT*.

### Protein production and purification

The pWarf(-)*Sa_siaT* plasmid was transformed into *E. coli* Lemo21(DE3) and grown in terrific broth media supplemented with kanamycin (50 μg/mL), chloramphenicol (34 μg/mL), L-rhamnose (100 μM), and induced with 0.4 mM isopropyl β-D-1-thiogalactopyranoside (IPTG) at 26°C overnight, with shaking at 180 rpm. For isothermal titration calorimetry and proteoliposome measurements, the protein was expressed in PASM-5052 auto-induction media (Lee et al., 2014). Cells were solubilized in phosphate-buffered saline (PBS), supplemented with cOmplete™ EDTA free protease inhibitor tablets (Roche), lysozyme (0.5 mg/mL), DNaseI (5 μg/mL), MgCl_2_ (2 mM) and lysed by sonication using a Hielscher UP200S Ultrasonic Processor at 70% amplitude in cycles of 0.5 s on, 0.5 s off, for 30 min. Cell debris was pelleted by centrifugation at 24,000 g, for 25 min, at 4°C and the cell membranes were collected by ultracentrifugation at 230,000 g, for 2 h, at 4°C and stored at −80°C until further use. Cell membranes were solubilized in 2% (w/v) n-dodecyl-ß-D-maltoside (DDM) for 2 h at 4°C and unsolublized material was removed by ultracentrifugation at 150,000 g. The protein was first purified using immobilized metal affinity chromatography; the supernatant was loaded onto a 5 mL HisTrap HP column (GE Healthcare) equilibrated with Buffer A (70 mM Tris-HCl, pH 8.0, 150 mM NaCl, 20 mM imidazole, 6% glycerol, 5 mM β-mercaptoethanol, and 0.0174% (w/v) DDM). The column was washed with Buffer A, followed by a 10% wash with Buffer B (Buffer A with 500 mM imidazole) and protein was eluted using 50% Buffer B. Protein was concentrated and simultaneously exchanged into Buffer C (50 mM Tris-HCl, pH 8.0, 150 mM NaCl, 0.0174% (w/v) DDM). For analytical ultracentrifugation experiments, Buffer C contained 0.174% DDM. The GFP-tag was cleaved with HRV3C protease in a 1:12.5 mass ratio (HRV3C:*Sa*SiaT) at 4°C for 18 h. Size exclusion chromatography was performed as a final purification step using a HiLoad 16/60 Superdex 200 column in Buffer C. Protein concentration was determined using a NanoDrop 1000 spectrophotometer at 280 nm, using an extinction coefficient of 75,750 M^−1^cm^−1^, and a molecular weight of 56.7 kDa following HRV3C cleavage of the GFP-tag.

### Analytical ultracentrifugation

Sedimentation velocity experiments were performed in a Beckman Coulter Model XL-I analytical ultracentrifuge equipped with UV/Vis scanning optics. Reference buffer solution (50 mM Tris-HCl, 150 mM NaCl, pH 8.0) and sample solutions (including reference buffer solution with 0.174% DDM, and *Sa*SiaT at four concentrations: 0.6, 0.4, 0.2, and 0.1 mg mL^−1^) were loaded into 12 mm double-sector cells with standard Epon 2-channel centerpieces and quartz windows. Cells were mounted in an eight hole An-50 Ti rotor and centrifuged at 50,000 rpm at 12°C. Interference and absorbance measurements at a wavelength of 280 nm were recorded over a radial position range of 5.8 to 7.3 cm within the cell, with measurements taken at sediment boundary intervals of 0.003 cm. The partial specific volume of *Sa*SiaT was calculated using SEDNTERP (Laue et al., 1992) and buffer density and buffer viscosity were experimentally measured with an Anton Paar DMA4100M density meter and Anton Paar Lovis 2000 ME microviscometer, respectively. The van Holde-Weischet and 2DSA-Monte Carlo analyses were performed using UltraScan III (Demeler and van Holde, 2004; Brookes and Demeler, 2007; Demeler and Brookes, 2007; Demeler, 2010).

### Bacterial growth experiment

*E. coli* strains JW3193 Δ*nanT*, JW3193 Δ*nanT_siaT, and E. coli* BW25113, which served as a wild type control, were grown (37°C, 250 rpm) overnight in low salt Luria-Bertani (LB) media. For Δ*nanT_siaT*, the LB media was supplemented with Zeocin™ (25 μg/mL). Overnight cultures were diluted to an OD_600_ of 0.05 and further grown (37°C, 250 rpm) in low salt LB media supplemented with 1 mM IPTG until they reached mid-logarithmic phase (OD_600_ of 0.35). Bacterial cultures were harvested by centrifugation (6,000 rpm, 10 min, 4°C), washed three times in M9 minimal media and diluted to an OD_600_ of 0.5. Cultures (20 μL) were added to a Costar Flat Bottom 96 well plate with lid containing M9 minimal media (180 μL) supplemented with Zeocin™ (25 μg/mL), IPTG (1 mM), thiamin hydrochloride (7 μM) and either *N*-acetylneuraminic acid (Neu5Ac, 4 mg/mL, 12.9 mM), *N*-glycolylneuraminic acid (Neu5Gc, 4 mg/mL, 12.3 mM) or glucose (0.4%) as the sole carbon source. In addition, bacterial growth was monitored in M9 minimal media without any carbon source. Growth at 37°C, with shaking at 250 rpm, was recorded at 600 nm every 10 min using the FLUOstar Omega microplate reader (BMG labtech). Growth curves represent the mean of four measurements ± standard error of the mean, or three measurements ± standard error of the mean for the control experiments.

### Microscale thermophoresis

The binding affinities for two sialic acids and purified *Sa*SiaT were determined using microscale thermophoresis. Experiments were performed on a Monolith NT.LabelFree instrument (NanoTemper Technologies) (Wienken et al., 2010; Soares da Costa et al., 2016; Stifter et al., 2018). Purified *Sa*SiaT was diluted to 2 μM in PBS buffer supplemented with 0.0174% (w/v) DDM, and incubated with Neu5Ac (from 0.3 μM to 10 mM), and Neu5Gc (0.08 μM to 2.5 mM), for 5 min prior to taking measurements. The samples were loaded into Monolith NT Standard Treated Capillaries (NanoTemper Technologies). Microscale thermophoresis measurements were carried out at 25°C using 20% LED power, and 20% microscale thermophoresis infrared laser power. The dissociation constants (*K*_*d*_) were determined using the mass action equation *via* the NT Analysis software version 1.5.41 (NanoTemper Technologies), using the signal from Thermophoresis + T-jump for triplicate experiments.

### Isothermal calorimetry

Purified *Sa*SiaT was concentrated to a final concentration of 170–240 μM using membrane ultrafiltration with a molecular weight cutoff of 50 kDa. The flow through was used to dilute 100 mM stock solutions of sialic acid to concentrations of 2.5–4 mM for Neu5Ac and 2–2.4 mM for Neu5Gc. Protein sample (206 μL) was loaded into the sample cell, and 70 μL of the respective sialic acid was loaded into the injection syringe. The system was equilibrated to 25°C with a stirring speed of 750 rpm. Titration curves were initiated by a 1 μL injection followed by 4 μL injections every 180 s. Background corrections were obtained by injection of sialic acids into buffer and buffer into protein with the same parameters. Biological triplicate experiments were analyzed using ORIGIN 7 with the first injection excluded. The curves were fitted into a single-site binding isotherm and *K*_*d*_ values were determined. Measurements were made using a Micro-200 Isothermal titration calorimeter or a PEAQ Isothermal titration calorimeter (MicroCal, Malvern).

### Sequence alignment and homology modeling of SiaT in an outward-facing open conformation

Multiple protein sequence alignment was performed between *Sa*SiaT and additional SiaT from various bacterial species, as described elsewhere (Wahlgren et al., 2018). This was used to compare conservation of the Neu5Ac binding site between organisms. A second multiple protein sequence alignment was performed between SiaT from 19 strains of *S. aureus*, to compare conservation of the Neu5Ac binding site between different isolates. These include *S. aureus* RF122, *S. aureus* ED133, *S. aureus* NR153, *S. aureus* XQ, *S. aureus* 93b_S9, *S. aureus* CFSAN007883, *S. aureus* HZW450, *S. aureus* MS4, *S. aureus* SA268, *S. aureus* SA40, *S. aureus* SA957, *S. aureus* M013, *S. aureus* FDA209P, *S. aureus* FDAARGOS_43, *S. aureus* NRS271, *S. aureus* MW2, *S. aureus* NRS143, *S. aureus* USA400-0051, *S. aureus* EDCC5464. Alignments were generated using *ClustalW* (Larkin et al., 2007).

The outward-facing open conformation of *Sa*SiaT was modeled on the outward-facing open structure of SiaT from *P. mirabilis* (*Pm*SiaT). These transporters share ~41% sequence identity. To build a homology model, an alignment between the two protein sequences was first generated using a global sequence alignment with *EMBOSS stretcher* (Myers and Miller, 1988). *MEDELLER* (Kelm et al., 2010) was used to create the model of *Sa*SiaT in the outward-facing open conformation using *Pm*SiaT (pdb entry 5nv9) as a template structure. Next, GROMACS 5.1.2 (Abraham et al., 2015) was used for energy minimization of the *Sa*SiaT homology model, using the GROMOS 54A7 force field. The resulting structure was superimposed onto the *Pm*SiaT structure with Neu5Ac bound using a Structural Alignment of Multiple Proteins (STAMP) structure-based sequence alignment in VMD MultiSeq. The structure was manually edited using COOT (without further energy minimization) to remove a clash between the sidechain of Tyr79 and the Neu5Ac. The sidechain was rotated to overlay with that of the corresponding residue (Phe78) in the template *Pm*SiaT structure. Other residues in the substrate binding site of the homology model were rotated to better represent the conformations found in the substrate bound *Pm*SiaT template.

### Proteoliposome assays

Purified *Sa*SiaT was reconstituted into proteoliposomes using a protocol previously optimized for *Pm*SiaT, with some modifications (Wahlgren et al., 2018). Briefly, 5 μg of protein was mixed with 120 μL 10% C_12_E_8_, 100 μL of 10% egg yolk phospholipids (w/v, sonicated as previously described to form liposomes, Scalise et al., 2014). Next, 20 mM of K^+^-gluconate buffered by 20 mM Tris-HCl, pH 7.0 was added to create a final volume of 700 μL. The mixture was incubated with 0.5 g Amberlite XAD-4 resin under rotatory stirring (1,200 rev/min) at 25°C for 40 min (Scalise et al., 2015). After reconstitution, 600 μL of proteoliposomes were loaded onto a Sephadex G-75 column (0.7 cm diameter × 15 cm height) pre-equilibrated with 20 mM Tris-HCl, pH 7.0, 40 mM sucrose to balance internal osmolarity. To generate a K^+^ diffusion potential, valinomycin (0.75 μg/mg phospholipid) prepared in ethanol was added to the proteoliposomes following Sephadex G-75 column chromatography, as previously described (Scalise et al., 2014; Wahlgren et al., 2018). As a control, ethanol was added to proteoliposomes, which did not exert any effect on the transport activity. After 10 s of incubation with valinomycin/ethanol, transport was started by adding 50 μM [^3^H]-Neu5Ac to the proteoliposomes in the presence of 25 mM NaCl. The initial rate of transport was measured by stopping the reaction after 5 min, i.e., within the initial linear range of [^3^H]-Neu5Ac uptake into the proteoliposomes. Transport was terminated once [^3^H]-Neu5Ac was removed by loading each proteoliposome sample (100 μL) on a Sephadex G-75 column (0.6 cm diameter × 8 cm height). Proteoliposomes were eluted with 1 mL 50 mM NaCl and collected in 4 mL of scintillation mixture, vortexed and counted. Radioactivity uptake in liposome controls (without incorporated protein) was negligible with respect to transport data. Uptake data were fitted in a first-order rate equation for time course plots, and kinetic data were fitted with a Michaelis-Menten or Hill equations. Non-linear fitting analysis was performed by Grafit software (version 5.0.13). To measure the specific activity of *Sa*SiaT, the amount of protein was estimated by NanoDrop. All measurements are presented as means ± SD from three independent experiments.

## Results and discussion

### Expression of *Sa*SiaT rescues an *E. coli* strain that lacks its endogenous sialic acid transporter

**Figure 1:**
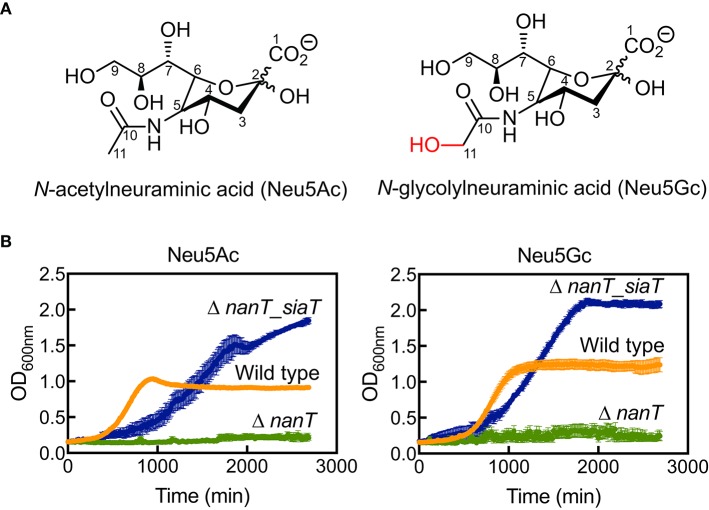
Bacterial growth experiments demonstrate *Sa*SiaT function. **(A)** The chemical structures of Neu5Ac and Neu5Gc. **(B)** Growth of *E. coli* wild type (orange), Δ*nanT* (green), and its complemented derivative Δ*nant*_*siaT* (blue) on Neu5Ac and Neu5Gc. While Δ*nanT* is unable to utilize Neu5Ac and Neu5Gc, *Sa*SiaT is able to rescue the growth of Δ*nanT* on both Neu5Ac and Neu5Gc sialic acids as the sole carbon source.

To demonstrate sialic acid transport by *Sa*SiaT, we first showed that *Sa*SiaT rescues the growth of an *E. coli* strain lacking the endogenous NanT sialic acid transporter (Δ*nanT*) when grown on Neu5Ac or Neu5Gc (Figure 1). The Neu5Ac and Neu5Gc differ by the addition of a hydroxyl at the C11 methyl of the *N*-acetyl group in Neu5Gc (Figure 1A). While *E. coli* JW3193 Δ*nanT* grows in M9 minimal media supplemented with glucose (Table 1), it is not able to utilize Neu5Ac or Neu5Gc as the sole carbon source (Figure 1B, data in green). After complementation of *E. coli* JW3193 Δ*nanT* with pJ422-01*Sa_siaT* (to produce *E. coli* JW3193 Δ*nanT_siaT*), the ability to grow on both sialic acids is restored (Figure 1B, data in blue). Notably, the growth rate of Δ*nanT_siaT* is faster when grown in M9 minimal media containing Neu5Gc as the sole carbon source, as opposed to Neu5Ac (Table 1). This could reflect more efficient transport of Neu5Gc, due to a higher affinity for Neu5Gc compared to Neu5Ac.

**Table 1:** Growth rates of the *E. coli* wild type, Δ*nanT* and Δ*nanT*_*siaT* in M9 minimal media containing different carbon sources.

**Strain**	**Neu5Ac**	**Neu5Gc**	**Glucose**	**No carbon source**
Wild type	3.56 (0.04) × 10^−3^	3.3 (0.1) × 10^−3^	5.5 (0.2) × 10^−3^	–
*ΔnanT*	–	–	4.3 (0.5) × 10^−3^	–
*ΔnanT*_*siaT*	1.3 (0.3) × 10^−3^	1.9 (0.05) × 10^−3^	3.86 (0.05) × 10^−3^	–

*Values represent the growth rate/min. A dash (–) indicates no growth. Values in brackets represent the standard error of measurement, where n = 4*.

Curiously, *E. coli* Δ*nanT_siaT* reached higher final optical density compared to wild type *E. coli* BW25113 (Figure 1B, data in blue compared to data in orange). This was unexpected, perhaps suggesting that the native *E. coli* NanT may be regulated in some way, thereby limiting sialic acid uptake. *E. coli* Δ*nanT_siaT* exhibits an extended lag-phase compared to wild type *E. coli* BW25113. Since SiaT expression was pre-induced with IPTG (see section Materials and Methods), it is likely that the growth lag is due to an increased metabolic burden caused by the overexpression of SiaT itself. This also explains the reduced growth rates of Δ*nanT_siaT* compared to the wild type (Table 1).

In short, we demonstrate that the *Sa*SiaT is functional *in vivo* and observe a preference for Neu5Gc in terms of maximum growth rate.

### Recombinant *Sa*SiaT is stably purified as a single species

**Figure 2:**
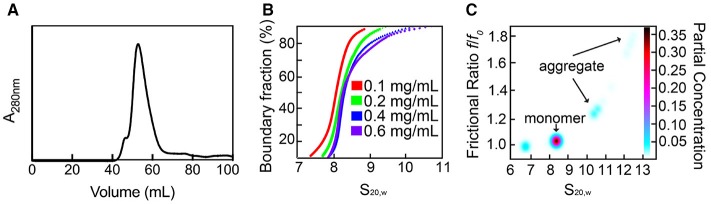
Recombinant *Sa*SiaT can be stably purified and occupies a predominantly single oligomeric state. **(A)** Size-exclusion chromatography trace of *Sa*SiaT at its final purification step. To the left of a main dominant peak there is a small shoulder, which may represent aggregate. **(B)** van Holde-Weischet sedimentation coefficient distributions show a dominant component at ~8 S. **(C)** 2DSA-Monte Carlo analysis of sedimentation velocity data of *Sa*SiaT at 0.6 mg/mL, as implemented by UltraScan III, shows a main peak comprising 70% of the signal.

*Sa*SiaT was successfully overexpressed, solubilized, and purified to homogeneity in buffer containing DDM detergent. The profile from size exclusion chromatography (Figure 2A) shows a dominant peak at ~55 mL, with a shoulder to the left that is consistent with a small amount of a larger component, possibly an aggregate. Analytical ultracentrifugation studies at protein concentrations ranging from 0.1 to 0.6 mg/mL were used to assess the stability and oligomeric state of purified recombinant *Sa*SiaT prior to functional studies. Analyses of absorbance data from analytical ultracentrifugation experiments, using van Holde-Weischet sedimentation coefficient distributions (Figure 2B), reveal a largely monodisperse solution with a major component at ~8 S. However, as evidenced by the shift of the distribution to the right, the van Holde-Weischet analysis also suggests that some protein may be aggregated. 2DSA-Monte Carlo analysis (Figure 2C) determines a major component at 8.2 S with a frictional ratio of 1.05, which is consistent with a molecular weight of 160 kDa (assuming a mass averaged v-bar of 0.76 for the detergent:protein complex). This is consistent with a *Sa*SiaT monomer surrounded by ~200 DDM molecules. This monomeric species represents ~70% of the total sample. The 2DSA-Monte Carlo analysis also shows a series of larger components ranging from 10.5 to 13.0 S with an increasing frictional ratio, that represent a small amount of aggregate. Overall, our studies demonstrate that *Sa*SiaT can be stably expressed and purified using the detergent DDM. Furthermore, when solubilized in DDM, it is largely a monomer that associates with ~200 DDM molecules in solution.

### Binding studies demonstrate that *Sa*SiaT has an altered specificity

**Figure 3:**
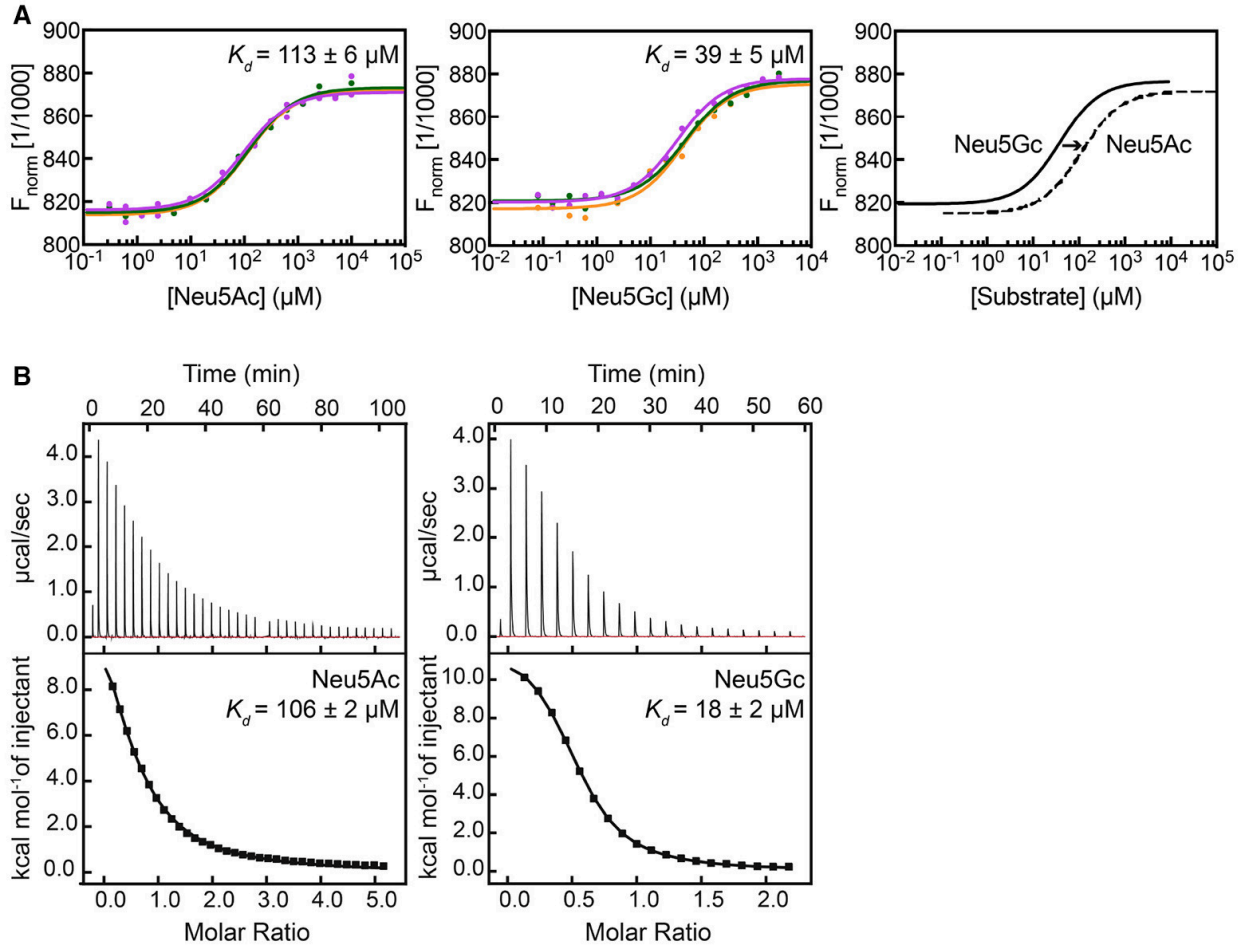
Orthogonal binding experiments demonstrate substrate ambiguity. **(A)** Microscale thermophoresis binding assay to measure the affinity of *Sa*SiaT for Neu5Ac and Neu5Gc sialic acids. Raw data are shown with the fit for three independent experiments with Neu5Ac (left) and Neu5Gc (middle). The *K*_*d*_ values are reported as the mean ± uncertainty in the mean of the fit using the signal from Thermophoresis + T-jump, from triplicate experiments, where *n* = 1 (we define *n* as the number of different recombinant protein preparations, which we view as equivalent to biological replicates). The *K*_*d*_ and associated error of each fit is given in Supplementary Table 2. The shift in *K*_*d*_ between both sialic acids is shown (right). *Sa*SiaT has a tighter affinity for Neu5Gc than Neu5Ac. **(B)** Representative isothermal titration calorimetry raw data (top panel) and binding isotherm (bottom panel) of one isothermal titration calorimetry experiment obtained by successive titration of Neu5Ac (left) or Neu5Gc (right) with purified *Sa*SiaT. The fit of a single binding site is shown in the bottom panels (black line). *K*_*d*_ values are reported as the mean ± SEM of the fit from three experiments using different protein preparations (*n* = 3).

Given the increased growth rate observed when grown on Neu5Gc compared to Neu5Ac, the binding affinity of Neu5Ac and Neu5Gc to recombinant *Sa*SiaT was determined using microscale thermophoresis (Figure 3A) and isothermal titration calorimetry (Figure 3B). By microscale thermophoresis, *Sa*SiaT has a considerably higher affinity for the Neu5Gc sialic acid (*K*_*d*_ = 39 ± 4 μM) compared to the Neu5Ac sialic acid (*K*_*d*_ = 113 ± 6 μM). Consistent with this, isothermal titration calorimetry experiments confirmed that *Sa*SiaT has a higher affinity for Neu5Gc (*K*_*d*_ = 18 ± 2 μM) than Neu5Ac (*K*_*d*_ = 106 ± 2 μM). Interestingly, *Pm*SiaT is the opposite, and displays a more similar, but higher binding affinity for Neu5Ac (*K*_*d*_^Neu5Ac^ = 58 ± 1 μM) compared to Neu5Gc (*K*_*d*_^Neu5Gc^ = 85 ± 2 μM) using microscale thermophoresis (Wahlgren et al., 2018). Thus, *Sa*SiaT reveals different substrate specificity compared to *Pm*SiaT.

### Three substitutions in the active site of *Sa*SiaT may explain altered substrate specificity

To reconcile at the molecular level the observed difference in substrate specificity, and ultimately, the difference between *Sa*SiaT and *Pm*SiaT, amino acid sequence analyses and homology modeling were used to examine the differences in the active site.

**Figure 4:**
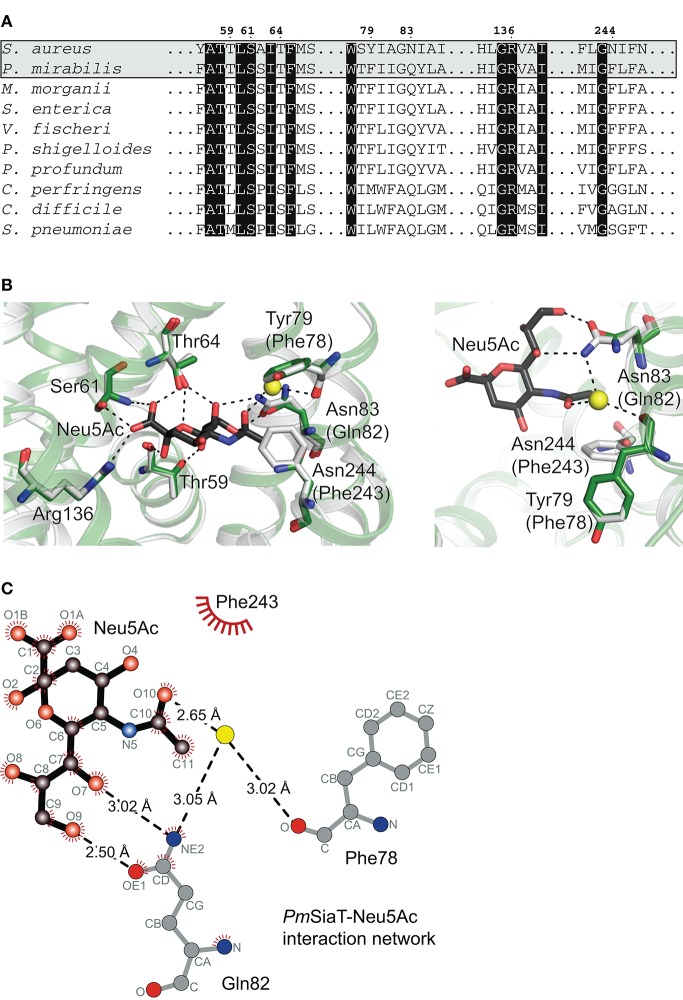
Sequence alignment and homology modeling probe substrate ambiguity. **(A)** Amino acid sequence alignment of *Sa*SiaT with SiaT transporters from eight additional bacterial species (Wahlgren et al., 2018). SiaT transporters from *S. aureus, P. mirabilis, Morganella morganii, S. enterica, Vibrio fischeri, Plesiomonas shigelloides, Photobacterium profundum, Clostridium perfringens, C. difficile*, and *Streptococcus pneumoniae* are aligned. Important residues in the Neu5Ac binding site in *Pm*SiaT (pdb entry 5nv9) are shown. Residues highlighted with black boxes are highly conserved, and important residues implicated in Neu5Ac binding in *Pm*SiaT are numbered according to *Sa*SiaT. **(B)** Superposition of the *Sa*SiaT homology model (green) and *Pm*SiaT (gray) with Neu5Ac bound (black). Residues are labeled according to *Sa*SiaT, with *Pm*SiaT in parentheses. A water molecule from *Pm*SiaT is shown in yellow. *Pm*SiaT coordinates are from pdb entry 5nv9. Black dashed lines depict hydrogen bonds, or a salt bridge with Arg136. On the right, the binding site has been rotated 90° and the substituted residues are shown. **(C)** The *Pm*SiaT-Neu5Ac interaction network (Wahlgren et al., 2018) with Gln82, Phe78, and Phe243 is represented as a Ligplot^+^ diagram (Laskowski and Swindells, 2011) using PDB entry 5nv9. Hydrogen bonds (dashed lines), hydrophobic contacts (arcs with spokes), and an interacting water molecule (yellow) are shown.

The Neu5Ac binding site, as determined in *Pm*SiaT (pdb entry 5nv9), is conserved among SiaT transporters from a number of bacterial species (Figure 4A) (Wahlgren et al., 2018). When comparing the sequences between *Sa*SiaT and *Pm*SiaT, there are three substitutions in *Sa*SiaT (*Pm*SiaT-Gln82 to *Sa*SiaT-Asn83, *Pm*SiaT-Phe78 to *Sa*SiaT-Tyr79, and *Pm*SiaT-Phe243 to *Sa*SiaT-Asn244, Figure 4A) that we predict are involved in substrate binding. These residues are highly conserved among *S. aureus* isolates (Supplementary Figure 2); they are, therefore, not specific to any particular isolate of *S. aureus*.

To map these substitutions within the putative active site, a homology model of *Sa*SiaT was built based upon the outward-facing open structure of *Pm*SiaT (pdb entry 5nv9) (Figure 4B). Superposition of the *Sa*SiaT homology model with *Pm*SiaT (r.m.s.d. = 0.172 Å for 364 α-carbon atoms) demonstrates that the *Pm*SiaT-Gln82 to *Sa*SiaT-Asn83, *Pm*SiaT-Phe243 to *Sa*SiaT-Asn244, and *Pm*SiaT-Phe78 to *Sa*SiaT-Tyr79 substitutions in *Sa*SiaT may be responsible for the altered substrate specificity observed in the binding experiments, since they are close to the C11 methyl group of the *N*-acetyl moiety of Neu5Ac (Figure 4B), which is hydroxylated in Neu5Gc.

In the *Pm*SiaT crystal structure, the side chain of Gln82 (*Sa*SiaT-Asn83) is in a position to form two hydrogen bonds with the hydroxyl group at C9 and the hydroxyl group at C7 of the Neu5Ac glycerol tail (Figure 4C) (Wahlgren et al., 2018). In *Sa*SiaT, the side chain of the Asn83 substitution is shorter than a Gln, which creates more space in the substrate-binding cavity. *Sa*SiaT-Asn83 may be in a position to bond the additional C11 hydroxyl of Neu5Gc (Figure 4C), but only if the C11 hydroxyl points toward the glycerol tail. Like *Pm*SiaT-Gln82, *Sa*SiaT-Asn83 is still in position to hydrogen bond the hydroxyl group at C9 of the glycerol tail, albeit with a longer hydrogen bond length.

The C10 carbonyl of the *N*-acetyl moiety in Neu5Ac is coordinated by the amide from the side chain of Gln82 through a water molecule, while the methyl group of the *N*-acetyl moiety of Neu5Ac is facing toward a hydrophobic patch formed by Phe78 and Phe243 (Figure 4C) (Wahlgren et al., 2018). In *Sa*SiaT, Tyr79, and Asn244 replace the equivalent positions of *Pm*SiaT-Phe78 and *Pm*SiaT-Phe243 (Figure 4B). *Sa*SiaT-Asn244 could facilitate a new interaction with the additional C11 hydroxyl group of Neu5Gc, and the side chain hydroxyl group of *Sa*SiaT-Tyr79 could create a more hydrophilic environment in the vicinity, which may be important for Neu5Ac and Neu5Gc discrimination.

There are other examples where the preference for Neu5Gc is mediated by new interactions, *via* hydrogen bonds, with the extra hydroxyl group present in Neu5Gc. A similar preference for Neu5Gc over Neu5Ac has been reported for the subtilase cytotoxin (SubAB) produced by Shiga-toxigenic *E. coli* (Byres et al., 2008), and the porcine rotavirus (Yu et al., 2012), both of which bind to glycans terminating with sialic acids. The crystal structure of SubB-Neu5Gc complex (pdb entry 3dwa) shows that the C11 hydroxyl group of the glycolyl in Neu5Gc forms important hydrogen bonds with the side chain of a Tyr, and the main chain of a Met (Byres et al., 2008). The crystal structure of the porcine rotavirus strain CRW-8 spike protein domain VP8 (pdb entry 3tay) has similar interactions between the glycolyl of Neu5Gc with the side chain of a Thr, and the main chain of a Tyr (Yu et al., 2012). Mutation of these residues results in a significant loss of activity. Similarly, the VP1 capsid protein from human polyomavirus 9 (HPyV9) has a preference for Neu5Gc over Neu5Ac (Khan et al., 2014). However, the VP1 capsid protein from a closely related homolog, monkey-derived simian B-lymphotropic polyomavirus (LPyV), has no such preference (Khan et al., 2014). Again, the preference for Neu5Gc is acquired by specific hydrogen bonds with the glycolyl of Neu5Gc, which LPyV cannot form.

In conclusion, we suggest that the altered specificity of *Sa*SiaT for Neu5Gc over Neu5Ac, compared to *Pm*SiaT, may be afforded by Asn substitutions at the 83 and 244 positions, and a Tyr substitution at position 78, in the substrate-binding site of *Sa*SiaT.

### Proteoliposome assays delineate the kinetics of sialic acid membrane transport

**Figure 5:**
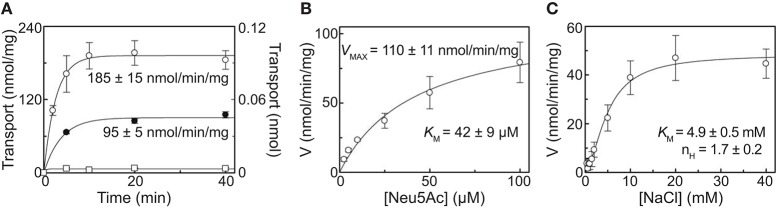
Proteoliposome assays demonstrate the ability to transport sialic acid, which is dependent on Na+. **(A)** Proteoliposome transport was started by adding 50 μM [^3^H]-Neu5Ac together with 25 mM NaCl to proteoliposomes reconstituted with purified recombinant *Sa*SiaT. In °, □, valinomycin was added to facilitate K^+^ movement prior to transport. In •, ethanol was added instead of valinomycin as a control. On the left Y-axis, specific transport activity is reported. In □, transport was measured in empty liposomes, with transport in empty liposomes reported on the right Y-axis. Transport was stopped at indicated times by passing proteoliposomes through Sephadex-G75 columns. Data were fitted to the first-order rate equation. **(B)** The transport of [^3^H]-Neu5Ac over a range of concentrations in the presence of 25 mM NaCl was measured in proteoliposomes reconstituted with purified recombinant *Sa*SiaT, with an imposed K^+^ diffusion membrane potential, over 5 min. Data were fitted to the Michaelis-Menten equation. **(C)** The transport of 50 μM [^3^H]-Neu5Ac in the presence of NaCl over a range of concentrations was measured in proteoliposomes reconstituted with purified recombinant *Sa*SiaT, with an imposed K^+^ diffusion membrane potential, over 5 min. Data were fitted to the Hill equation. All data are presented as mean ± SD from three independent experiments.

To demonstrate the ability of purified recombinant *Sa*SiaT to not only bind sialic acids, but to also transport them across a lipid membrane, we reconstituted the protein into proteoliposomes using native *E. coli* lipids and measured time dependent uptake of [^3^H]Neu5Ac (Figure 5A). The transporter mediated a Na^+^-dependent uptake of [^3^H]Neu5Ac, stimulated by an imposed membrane potential. Similar to *Pm*SiaT (Wahlgren et al., 2018), in the presence of an imposed membrane potential, transport at equilibrium was almost doubled (185 ± 15 nmol/min/mg) compared with transport in the absence of an imposed membrane potential (95 ± 5 nmol/min/mg) (Figure 5A). Transport of [^3^H]Neu5Ac in liposomes without *Sa*SiaT protein was negligible with respect to reconstituted *Sa*SiaT proteoliposomes.

In optimal proteoliposome transport conditions with an imposed membrane potential, [^3^H]Neu5Ac is transported by *Sa*SiaT with a *K*_M_ of 42 ± 9 μM and a *V*_max_ of 110 ± 11 nmol/min/mg (Figure 5B). This is lower (a higher binding affinity) than the measurements made using microscale thermophoresis and isothermal titration calorimetry, which may reflect the altered solubilization of the transporter in lipid, as opposed to detergent. The *K*_M_ for [^3^H]Neu5Ac transport by *Sa*SiaT is almost twice that of *Pm*SiaT (Wahlgren et al., 2018). Consistent with the binding experiments, the molecular basis of this difference is likely that *Sa*SiaT has a lower affinity for Neu5Ac compared to *Pm*SiaT.

The kinetics of Na^+^ transport by *Sa*SiaT was measured in proteoliposomes, giving a *K*_M_ of 4.9 ± 0.5 mM (Figure 5C). As demonstrated by the cooperativity index calculated from the Hill plot, the transport stoichiometry is more than one for Na^+^. This is in the same order calculated for *Pm*SiaT, which transport two Na^+^ for every Neu5Ac (Wahlgren et al., 2018).

To conclude, proteoliposome experiments demonstrate that the recombinant *Sa*SiaT is functional and able to transport Neu5Ac, that an electrogenic gradient drives transport, that the affinity for Neu5Ac is less than for *Pm*SiaT, and that two Na^+^ ions are transported for every sialic acid.

## Conclusions

Overall, we demonstrate that *Sa*SiaT is a functional sialic acid transporter, with a considerably higher binding affinity for Neu5Gc over Neu5Ac. Compared to *Pm*SiaT, in which Neu5Gc and Neu5Ac have similar binding affinities (Wahlgren et al., 2018), *Sa*SiaT has altered substrate specificity. We propose that three residues unique to the *Sa*SiaT substrate-binding site (Tyr79, Asn83, and Asn244) achieve a higher affinity to Neu5Gc. Like SubAB, the porcine rotavirus, and HPyV9, which also have a preference for Neu5Gc over Neu5Ac (Byres et al., 2008; Yu et al., 2012; Khan et al., 2014), specific hydrogen bonds with the C11 hydroxyl of Neu5Gc, and a hydrophilic environment in the vicinity of the Neu5Gc glycolyl chain, afford this specificity (Khan et al., 2014).

Although humans cannot synthesize Neu5Gc, they can acquire it from red meat and milk in the diet (Varki, 2010; Varki et al., 2011). Consequently, metabolic incorporation of Neu5Gc has been identified in the human gut epithelium and kidney vasculature (Tangvoranuntakul et al., 2003; Byres et al., 2008; Banda et al., 2012). Since some bacteria and viruses can discriminate between sialic acid variants (Byres et al., 2008; Yu et al., 2012; Khan et al., 2014; Stencel-Baerenwald et al., 2014), this could, in turn, influence their host or tissue range. It is possible that the ability of *Sa*SiaT to bind Neu5Gc with higher affinity (compared to Neu5Ac) confers an advantage to *S. aureus* in specific niches.

Because a functional sialic acid transporter is essential for the uptake and utilization of sialic acid in a range of pathogenic bacteria, these transporters present a new avenue for drug design. The work presented here underpins the development of inhibitors that target SiaT transporters, and in particular, *S. aureus*.

## Author contributions

RF and RD conceived the project. Cloning and expression trials of *Sa*SiaT were carried out by ED and SR. Large-scale expression, membrane preparation, and protein purification were carried out by ED, WW, DR, and RN. Experiments for whole-cell functional analysis were designed by EC, ED, RN, and DR, and carried out by RN and DR. DR analyzed the results. Analytical ultracentrifugation experiments were performed and analyzed by SK. Proteoliposome assays were designed by MS, WW, RD, and CI. MS conducted and analyzed these experiments. Microscale thermophoresis experiments were carried out and analyzed by RN, TS, and MP. Isothermal titration calorimetry was performed and analyzed by WW. RN, WW, and JA carried out homology modeling and analysis of *Sa*SiaT. RN, WW, DR, MS, SK, CI, JA, RF, and RD wrote the manuscript, while all authors discussed the results and made manuscript revisions.

### Conflict of interest statement

The authors declare that the research was conducted in the absence of any commercial or financial relationships that could be construed as a potential conflict of interest.
