# Supplementary material for: The Sodium Sialic Acid Symporter From Staphylococcus aureus Has Altered Substrate Specificity
Source: Front Chem. 2018 Jul 4;6:233. doi: 10.3389/fchem.2018.00233 (PMC6039549; doi:10.3389/fchem.2018.00233)
Supplement: Supplementary file 1 [file Table_1.PDF]

The sodium sialic acid symporter from *Staphylococcus aureus* has altered substrate specificity

Rachel A. North[1,2,3†], Weixiao Yuan Wahlgren[3,4†], Daniela M. Remus[1,2], Mariafrancesca Scalise[5], Sarah A. Kessans[1,2], Elin Dunevall[3], Elin Claesson[3], Tatiana P. Soares da Costa[6], Matthew A. Perugini[6], S. Ramaswamy[7], Jane R. Allison[2,8,9], Cesare Indiveri[5], Rosmarie Friemann [3,4*], Renwick C. J. Dobson [1,10*]

Supplementary Information

```
siaT            ATGAAAGAAGTTGGATTTGGCACACTGAACTGGGTTGCCGTTATCATTTATCTACTAGCTATGTTGTTCA
Optimized       ATGAAAGAAGTTGGTTTTGGCACCCTGAATTGGGTTGCAGTTATTATCTATCTGCTGGCCATGCTGTTTA
                ************** ******** ***** ******** ***** ** ***** ** ** *** **** *

siaT            TTGGCGTTTATTTTACCAAGCGCGCGAGCCAAAGTACGAATAGTTTCTTTACCGCAAGTGGTCGCTTGCC
Optimized       TCGGTGTGTATTTTACCAAACGTGCAAGCCAGAGCACCAATAGCTTTTTTACCGCAAGCGGTCGTCTGCC
                * ** ** *********** ** ** ***** ** ** ***** ** *********** *****  ****

siaT            ATCTTGGGTAGTTGGCTTTTCAATTTATGCCACTACATTAAGTGCAATTACATTTATGTCTACACCAGAG
Optimized       GAGCTGGGTTGTTGGTTTTAGCATTTATGCAACCACCCTGAGCGCAATTACCTTTATGAGCACACCGGAA
                    ***** ***** ***   ******** ** **  * ** ******** ******   ***** **

siaT            AAAGCATTTTTAACAGATTGGTCATATATTGCAGGTAACATCGCTATCGTAGCAATTATTCCATTACTCA
Optimized       AAAGCATTTCTGACCGATTGGAGCTATATTGCAGGTAATATTGCCATTGTTGCCATTATTCCGCTGCTGA
                ********* * ** ******   ************** ** ** ** ** ** ********  * ** *

siaT            TTTATTTCTATGTTCCATTCTTTAAAAAGTTAAAGGTAACATCTGCATATGAATATCTAGAAGCACGTTT
Optimized       TCTATTTCTATGTGCCGTTCTTCAAAAAACTGAAAGTTACCAGCGCCTATGAATATCTGGAAGCACGTTT
                * *********** ** ***** *****  * ** ** **    ** *********** ***********

siaT            CGGACCTAGCATTCGTGTCATTGGCTCTTTACTATTTGTAGTTTATCATCTAGGGCGTGTAGCAATCGTT
Optimized       TGGTCCGAGCATTCGTGTTATTGGTAGCCTGCTGTTTGTTGTTTATCATCTGGGTCGTGTTGCCATCGTT
                 ** ** *********** *****     * ** ***** *********** ** ***** ** ******

siaT            ATCTACTTACCAACATTAGCGATTACATCTGTATCAGACATGAATCCTTATATCGTTGCATCACTCGTTG
Optimized       ATTTATCTGCCGACCCTGGCAATTACCAGCGTTAGCGATATGAATCCGTATATTGTTGCAAGCCTGGTTG
                ** **  * ** **  * ** *****    **    ** ******** ***** ******   ** ****

siaT            GTTTACTATGTATTTTATATACATTTTTAGGTGGATTCGAAGGTGTGGTTTGGAGTGATTTCATTCAAGG
Optimized       GTCTGCTGTGTATTCTGTATACCTTTCTGGGTGGTTTTGAAGGTGTTGTTTGGAGCGATTTTATTCAGGG
                ** * ** ****** * ***** *** * ***** ** ******** ******** ***** ***** **

siaT            CGTCATTTTATTAGGCGGCGCTTTAGTTATCATTATTCTAGGTGTTATGAACATTAAAGGCGGTTTTGGC
Optimized       CGTTATTCTGCTGGGTGGTGCACTGGTTATCATTATTCTGGGTGTGATGAACATCAAAGGTGGCTTTGGC
                *** *** *  * ** ** **  * ************** ***** ******** ***** ** ******

siaT            ACTGTCTTTGCAGATGCGATTGAGCACAAAAAATTAATTAGTGCAGACAATTGGAAACTAAATACTGCAG
Optimized       ACCGTTTTTGCAGATGCAATTGAACATAAAAAACTGATCAGCGCAGACAACTGGAAACTGAATACCGCAG
                ** ** *********** ***** ** ****** * ** ** ******** ******** ***** ****

siaT            CAGCTGCCATTCCAATTATTTTCCTAGGAAATATTTTCAACAACTTGTATCAATACACGGCGAGTCAAGA
Optimized       CAGCAGCAATTCCGATTATCTTTCTGGGCAACATTTTCAACAACCTGTATCAGTATACCGCCAGCCAGGA
                **** ** ***** ***** ** ** ** ** ************ ******* ** ** ** ** ** **

siaT            CGTCGTGCAACGTTATCAAGCTTCTGATAGTTTAAAAGAAACAAATAAATCGTTATGGACAAATGGTATC
Optimized       TGTTGTTCAGCGTTATCAGGCAAGCGATAGCCTGAAAGAAACCAATAAAAGCCTGTGGACCAATGGTATT
                 ** ** ** ******** **    *****  * ******** ******    * ***** ********

siaT            CTAGCTTTAATTTCAGCACCCTTATTTTATGGTATGGGTACAATGTTGTATTCATTTTATGCACATGAAG
Optimized       CTGGCACTGATTAGCGCACCGCTGTTTTATGGTATGGGCACCATGCTGTATAGCTTTTATGCACATGAAG
                ** **  * ***   *****  * ************** ** *** *****   ****************

siaT            CTGTTTTACCAAAAGGCTTCAATACATCATCTGTAGTGCCATATTTCATTTTGACTGAGATGCCACCATT
Optimized       CAGTTCTGCCGAAAGGTTTTAATACCAGCAGCGTTGTTCCGTATTTTATCCTGACCGAAATGCCTCCGTT
                * *** * ** ***** ** *****       ** ** ** ***** **  **** ** ***** ** **

siaT            TGTAGCAGGATTACTTATTGCAGCCATTTTCGCCGCTGCACAGTCTACCATTTCATCTAGTTTAAATTCT
Optimized       TGTTGCAGGTCTGCTGATTGCAGCAATTTTTGCAGCAGCACAGAGTACCATTAGCAGCAGCCTGAATAGC
                *** *****  * ** ******** ***** ** ** ******  *******      **  * ***

siaT            ATATCTGCTTGTATTTCAATCGACATTAAGCAACGCTTCTTCGGAAAAGGTAGCGAGCGACACGAAGTTA
Optimized       ATTAGCGCATGTATTAGCATCGATATCAAACAGCGCTTTTTTGGTAAAGGTAGCGAACGTCATGAAGTGA
                **    ** ******   ***** ** ** ** ***** ** ** *********** ** ** ***** *

siaT            ACTTTGCTCGTTTCATTATCATCATTGCAGGTATTTTCGGTTTTGGAATGTCACTATACTTAATTGCTTC
Optimized       ATTTTGCCCGTTTCATTATTATCATTGCCGGTATCTTTGGCTTTGGTATGAGCCTGTATCTGATTGCCAG
                * ***** ***********

siaT            TAATTCAAATGACTTATGGGATTTATTCTTGTTTGTGACTGGATTATTCGGCGTTCCATTGGCTGGTGTA
Optimized       TAATAGCAATGATCTGTGGGACCTGTTTCTGTTTGTTACCGGTCTGTTTGGTGTTCCGCTGGCAGGCGTT
                ****   *****  * *****  * **  ******* ** **  * ** ** *****  **** ** **

siaT            TTTGCAGTTGGTATTTTCACTAAACGTACGAATACATTCGGTGTTATTTGTGGATTAATATTGGGTATCA
Optimized       TTTGCCGTTGGTATTTTTACAAAACGTACCAATACCTTTGGCGTGATTTGTGGTCTGATTCTGGGTATTA
                ***** *********** ** ******** ***** ** ** ** ********  * **

siaT            TCTTTGCTTACGTCTATAATGGTGTTGGCAAAGGTAACTCACCTTTCTATGTATCTACCATTTCATTTAC
Optimized       TCTTCGCCTATGTTTATAATGGTGTGGGCAAAGGTAATAGCCCGTTTTATGTTAGCACCATCAGCTTTAC
                **** ** ** ** *********** ***********    ** ** *****    *****    *****

siaT            AGTTGCTTTTGTCTTTGCTTATATACTTAGCTTCATTGTCCCTTCAAAACATAAAAAAGATATAACGGGA
Optimized       CGTTGCCTTTGTTTTTGCATATATCCTGAGCTTTATTGTGCCGAGCAAACACAAAAAAGATATTACGGGT
                 ***** ***** ***** ***** ** ***** *****

siaT            TTAACAATTTTCGAAAAAGATAAACCATCAACATACATTTCAAAAACGGCTACGAAAAAGTAG
Optimized       CTGACCATCTTTGAAAAAGATAAACCGAGCACCTACATTAGCAAAACCGCAACCAAAAAATGA
                 * ** ** ** **************    ** ******   ***** ** ** ***** *
```

Supplementary Figure 1. Nucleotide alignment of *siaT* from *S. aureus* RF122 and the codon optimized sequence for *E. coli*. Asterisks indicate identical nucleotides.Alignments were generated using *ClustalW* (Larkin et al., 2007).

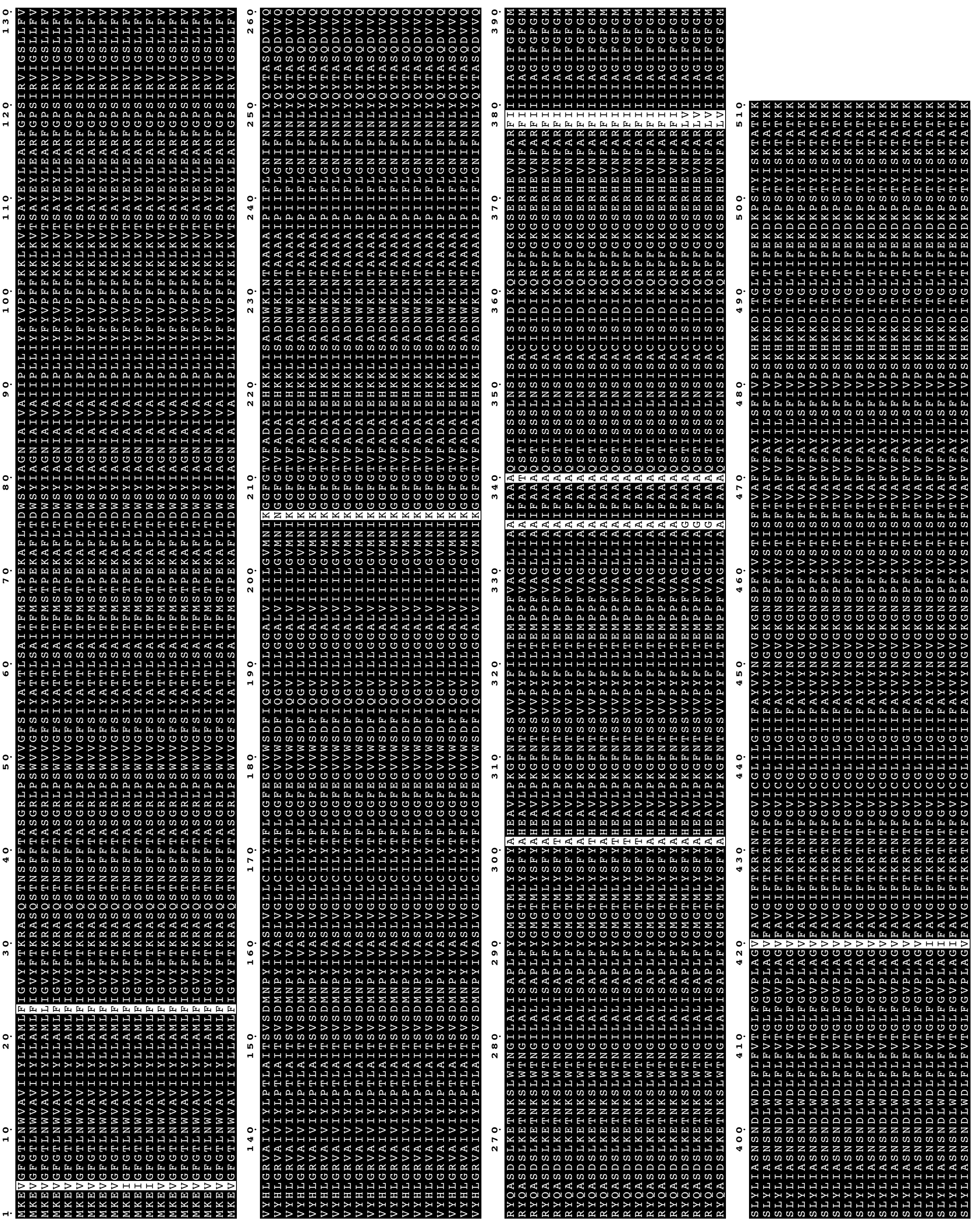

Supplementary Figure 2. Amino acid sequence alignment of SiaT from 19 *S. aureus* isolates. Residues highlighted with a black box are highly conserved. Residues are numbered according to *S. aureus* RF122.

Supplementary Table 1 | Oligonucleotide sequences for PCR amplification of *S. aureus siaT*. *Sa_siaT*-F1 and *Sa_siaT*-R1 were used for subsequent cloning into pWarf(-). *Sa_siaT*-F2 and *Sa_siaT*-R2 were used for subsequent cloning into pJ422-01.

**Primer name**	**Oligonucleotide sequence (5' – 3')**
Sa_siaT-F1	aagaaggagactcgagatgaaagaagttggttttggc
Sa_siaT-R1	agacttccaaggatcctttttggttgcggttttgc
Sa_siaT-F2	taggaggtaaacataatgaaagaagttggttttggcaccc
Sa_siaT-R2	ggcctgtacagaatttcatttttggttgcggttttgc

Supplementary Table 2 | Microscale thermophoresis binding assay to measure the affinity of *Sa*SiaT for Neu5Ac and Neu5Gc. The K_d values and associated error, calculated using the mass action equation *via* the NT Analysis software version 1.5.41 (NanoTemper Technologies), using the signal from Thermophoresis + T-jump for three replicates.

**Replicate**	**Neu5Ac**	**Neu5Gc**
1	101 ± 4	30.9 ± 0.9
2	116 ± 4	45 ± 2
3	121 ± 4	40 ± 2
